# General Resume of the Sick and Wounded of the Confederate States Army under Treatment during the Years 1861 and 1862

**Published:** 1866-07

**Authors:** 


### *General Resume of the Sick and Wounded of the Confederate States Army under Treatment during the years* 1861 *and* 1862.

From all the reports filed in the Surgeon-General's office for the years 1861 and 1862, exclusive of the few scattering ones which reached us from the Trans-Mississippi department, we are enabled to sum up the sickness and mortality occurring in the Southern armies during the late war as follows:

*Continued Fevers.*—Field reports, 36,746 cases and 5,205 deaths. Hospital reports, 40,565 cases and 7,020 deaths.

*Paroxysmal Fevers.*—Field reports, 115,415 cases and 848 deaths. Hospital reports, 49,311 cases and 485 deaths.

*Eruptive Fevers.*—Field, 44,438 cases and 1,036 deaths. Hospitals, 32,755 cases and 1,238 deaths.

*Diarrhœa and Dysentery.*—Field, 226,828 cases and 1,696 deaths. Hospitals, 86,506 cases and 1,658 deaths.

*Pulmonary Affections.*—Field, 42,204 cases, 3,535 deaths, and 4,538 discharges from service. Hospitals, 36,988 cases, 4,538 deaths, and 1,135 discharges.

*Rheumatism.*—Field, 29,334 cases, 1,142 discharges. Hospitals, 30,438 cases and 700 discharges.

*Gun-Shot Wounds.*—Field, 29,569 cases, 1,623 deaths and 493 discharges. Hospitals, 48,724 cases, 2,618 deaths, and 472 discharges. Killed in battle, 8,087.

*All other Diseases.*—Field, 324,321 cases and 2,278 deaths. Hospitals, 123,402 cases and 1,802 deaths.

Whole number of cases exhibited in the field reports during 1861 and 1862, was 848,555; of which 16,220 died, and 10,455 were discharged from service. There were admitted in hospitals for the same period 447,689 cases: of which 19,359 died, and 6,485 were discharged. Total deaths in two years, 35,579.

COMPOUND FRACTURE OF THE THIGH TREATED WITHOUT AMPUTATION.

| | Recoveries | Deaths.... | Days..... | Inches.... |
|---|---|---|---|---|
| | 116 | 105 | 000 | 000 |
| Average period of recovery | 000 | 000 | 104 | 000 |
| Greatest period of recovery | 000 | 000 | 255 | 000 |
| Least period of recovery | 000 | 000 | 41 | 000 |
| Average period of death | 000 | 000 | 52 | 000 |
| Greatest period of death | 000 | 000 | 185 | 000 |
| Least period of death | 000 | 000 | 1 | 000 |
| Average amount of shortening | 000 | 000 | 000 | 1.9 |
| Greatest amount of shortening | 000 | 000 | 000 | 5.0 |
| Least amount of shortening | 000 | 000 | 000 | 0.5 |

[*Richmond Medical Journal, from Confederate States Medical Journal.*

—A curious effect of the influence of civilization upon nature is seen in Pennsylvania. The Flora of the State is found to have undergone remarkable changes, plants that were formerly rare, being now quite abundant. This effect is attributed to the spread of railways, and the change is so marked that some botanists think the "foreign" Flora will supplant the native. The valley of the Susquehanna has already been taken possession of by the invaders.

*Ozone.*—M. Soret has determined that the density of ozone is one and a half times greater than that of oxygen. Dr. Boeckel, of Strasburg, has shown from observations conducted during a period of eleven years:

1. That there is more ozone in the spring of the year.
2. That May is the richest month.
3. That October and November are the poorest.
4. That there is less ozone at night than during the day.
5. That certain years were rich in ozone—1852, 1863.
6. That the barometric variations, morning and evening, coincided with the variations in the quantities of ozone.

*Medical Statistics.*—The Secretary of War, in compliance with a resolution of the Senate, calling for a compendium of the medical statistics collected during the war, states, on the authority of the Surgeon-General, that the records of many of the hospitals have not been received, and that the tabulation of those at hand is not complete; so that any compendium of the medical statistics of the war, at this time, must necessarily be based upon partial data, and hence be untrustworthy and valueless.

— A great many examinations of air are now making by scientific men. The very worst atmosphere yet found was in a court-room, the specimen showing that 5,000 parts of oxygen were absent in each 1,000,000 of air. To have 1,000 parts gone is bad, and 2,000 parts very bad. The only parallel to this court-room was found in the under galleries of coal mines.

